# Influence of schooling on language abilities of adults without linguistic disorders

**DOI:** 10.1590/S1516-31802009000300005

**Published:** 2009-10-06

**Authors:** Ellen Cristina Siqueira Soares, Karin Zazo Ortiz

**Affiliations:** 1 MSc. Doctoral student at Speech Pathology Department, Universidade Federal de São Paulo (Unifesp), São Paulo, Brazil.; 2 PhD. Professor at Speech Pathology Department, Universidade Federal de São Paulo (Unifesp), São Paulo, Brazil.

**Keywords:** Language, Educational status, Language tests, Evaluation studies [Publication type], Adult, Linguagem, Escolaridade, Testes de linguagem, Estudo de avaliação, Adulto

## Abstract

**CONTEXT AND OBJECTIVE:**

In order to properly assess language, sociodemographic variables that can influence the linguistic performance of individuals with or without linguistic disorders need to be taken into account. The aim of this study was to evaluate the influence of schooling and age on the results from the Montreal Toulouse (Modified MT Beta-86) language assessment test among individuals without linguistic disorders.

**DESIGN AND SETTING:**

Cross-sectional study carried out between March 2006 and August 2007 in the Speech, Language and Hearing Pathology Department of Universidade Federal de São Paulo (Unifesp), São Paulo, Brazil.

**METHODS:**

Eighty volunteers were selected. Schooling was stratified into three bands: A (1-4 years), B (5-8 years) and C (nine years and over). The age range was from 17 to 80 years. All the subjects underwent the Montreal Toulouse (Modified MT Beta-86) language assessment protocol.

**RESULTS:**

Statistically significant differences were found in relation to schooling levels, in the tasks of oral comprehension, reading, graphical comprehension, naming, lexical availability, dictation, graphical naming of actions and number reading. Statistically significant age-related differences in dictation and lexical availability tasks were observed.

**CONCLUSIONS:**

The Montreal Toulouse (Modified MT Beta-86) test seems to be sensitive to variations in schooling and age. These variables should be taken into account when this test is used for assessing patients with brain damage.

## RESUMO

**CONTEXTO E OBJETIVO:**

Para que a avaliação de linguagem seja adequada, faz-se necessário verificar a influência de variáveis sócio-demográficas que possam interferir no desempenho linguístico de indivíduos sem queixas linguísticas e de sujeitos com alterações de linguagem. O objetivo do estudo foi avaliar a influência da escolaridade e da idade no resultado do teste de avaliação de linguagem de Montreal Toulouse (MT Beta-86 Modificado) em pessoas sem queixas linguísticas.

**TIPO DE ESTUDO E LOCAL:**

Estudo transversal, desenvolvido entre março de 2006 a agosto de 2007, no Departamento de Fonoaudiologia da Universidade Federal de São Paulo.

**MÉTODOS:**

Foram selecionados 80 sujeitos voluntários. A escolaridade variou em três faixas: A (1-4 anos), B (5-8 anos) e C (9 anos e acima). A idade variou entre 17 e 80 anos. Todos os sujeitos foram submetidos ao protocolo de avaliação de linguagem Montreal Toulouse (MT Beta-86 Modificado).

**RESULTADOS:**

Foram verificadas diferenças estatisticamente significantes segundo a escolaridade nas tarefas de compreensão oral, leitura, compreensão gráfica, denominação, disponibilidade lexical, ditado, denominação gráfica de ações e leitura de números. Em relação à idade, verificamos diferenças estatisticamente significantes nas tarefas de ditado e disponibilidade lexical.

**CONCLUSÕES:**

As provas do teste Montreal Toulouse (MT Beta-86 Modificado) parecem ser sensíveis à variação de escolaridade e idade. Estas variáveis devem ser levadas em consideração quando o teste é utilizado para avaliação de sujeitos com lesão cerebral.

## INTRODUCTION

Language assessment among adults with acquired brain lesions entails characterizing oral production at the phonetic-phonological, syntactic-semantic, pragmatic and discursive levels. Graphical performance should be characterized according to graphemic, syllabic, lexical, syntactic, semantic, pragmatic and discursive levels, and oral and graphical comprehension in terms of words, phrases and stories. Guidelines recommend the use of tests to carry out this appraisal.^1^

Tests enable comparisons between the performance of subjects with brain lesions and that of healthy subjects who have the same schooling level, or allow intra-subject comparisons among brain lesion patients when tests are used longitudinally.

Assessment of language and communication abnormalities in brain lesion patients can fulfill clinical requirements by detecting and diagnosing any such disorders, while providing answers to issues such as brain and language interrelationships. Administration of language tests must take into account certain aspects of the test subjects, such as inexperience with tests, anxiety and degree of familiarity with the examiner or the situation, when interpreting results. This holds particularly true when individuals of low education level or elderly patients are involved.^2^

The first language assessment tests for adults emerged in 1935. The most well-known batteries include the Boston Diagnostic Aphasia Examination,^3^ Minnesota Test for Differential Diagnosis of Aphasia,^4^ Porch Index of Communication Abilities,^5^ Western Aphasia Battery^6^ and Token Test.^7^

Since language assessment is a key element in neuropsychological assessment, it is important for the protocols in use to be widely known. This is especially so when they are used in countries with sociocultural diversity, including the numbers of years of schooling and different educational levels attained. In such cases, it is extremely important to investigate the differences in language abilities that might exist within the normal population and find a profile that could be used for language assessment among brain-damaged patients.

Neuropsychological assessment instruments with norms for the Brazilian population remain scarce. In a bid to adopt an assessment protocol containing prompts that reflect everyday situations and linguistic realities as closely as possible, the performance of normal subjects on the Montreal Toulouse (Modified MT Beta-86) Test has been studied and characterized. Given that the Montreal Toulouse (Modified MT Beta-86) test was originally devised in the Latin language of Portuguese, it offers advantages over language tests that have been adapted from originals in the Anglo-Saxon languages.

The Montreal Toulouse (Modified MT Beta-86) is made up of tests that can characterize oral and graphical production, assess oral and graphical comprehension and measure repetition and fluency. This test was devised to assess the changes in language found among aphasic subjects.

Application of the test to normal subjects may make it possible to identify the aspects of the test that are influenced by schooling and age. From this, scores for use in language assessments on aphasic subjects can be obtained.

## OBJECTIVE

The aim of the present study was to ascertain the impact of sociocultural factors such as the number of years of schooling and age, on the performance of subjects without language deficits in the Montreal Toulouse (Modified MT Beta-86) test.

## METHODS

This was a cross-sectional study approved by the Universidade Federal de São Paulo (Unifesp) Research Ethics Committee (protocol no. 0151/05).

The inclusion criteria were that the subjects should be more than 17 years old, with no language deficits, while the exclusion criteria were diagnoses or histories of hearing, psychiatric and/or neurological disorders, based on a previously administered questionnaire.

All subjects were given information regarding the study. They underwent language assessment using the Montreal Toulouse (Modified MT Beta-86) test, after having signed the free and informed consent statement. The assessment protocol was administered by the researcher in a session lasting one hour and 30 minutes.

The sample size calculation for this study was n = [(Z_a/2_ x s)/E]2, taking a 5% significance level, a standard deviation of 7.5 according to a comparative study between different schooling levels^8^ and a standard error of 2.0. This calculation yielded a minimum sample size for this study of 54 individuals.

Eighty volunteers were selected from among the individuals accompanying patients who were attending the Acquired Speech and Language Neurological Disturbances outpatient unit and other clinics within the Department of Speech, Language and Hearing Pathology, Unifesp. The gender distribution of the final sample was 47 women and 23 men. The breakdown of the sample according to schooling was 22 in group A (1-4 years of education), 24 in group B (5-8 years) and 34 in group C (nine years and over).

The Montreal Toulouse (Modified MT Beta-86) test includes two books containing pictograph and written prompts (drawings/illustrations), along with five tangible objects (comb, key, ashtray, cup and paper), as well as an individual sheet for noting down answers. The objective of the test is to ascertain speech and language manifestations and to determine the type of aphasia presented.

The current study aimed to investigate normal individuals’ performance, as follows:

1. Guided interview: The subject had to answer 12 open questions, some of which subdivided. The subtests assessed both oral production and oral comprehension. For this study, oral comprehension was scored.
2. Reciting or automatisms: The task was made up of natural series (numbers, months of the year and days of the week). One point was given for each series produced correctly.
3. Oral comprehension: This task involved a total of 42 items assessing oral comprehension of words, along with simple and complex phrases. The subjects provided responses to a naming task.
4. Repetition: The subject had to repeat a total of 33 items, comprising words, non-words and phrases.
5. Reading: The subject had to read a total of 33 prompts such as words, non-words and phrases.
6. Written comprehension: 16 boards containing four to six pictures were presented, along with 16 written cards that the subject had to read and match with the corresponding graphical prompt. There were boards representing words or simple and complex phrases.
7. Naming: The subject had to name 31 pictures.
8. Lexical availability: The subject had to produce the greatest number of animals they could within a 60-second interval, while the investigator recorded the number of words produced. Each item scored one point. The task was timed using a stopwatch.
9. Buccofacial praxis: the subject had to make six praxic non-verbal gestures, elicited by verbal command.
10. Naming of body parts (oral): The subject had to understand and indicate on themselves, eight body parts named by the investigators.
11. Handling of objects following verbal instructions: the subject had to understand and carry out eight commands given by the investigator, using concrete objects (key, comb, cup, ashtray and paper). Phrase complexity gradually increased.
12. Copying: The subject had to copy three words and one phrase.
13. Dictation: The subject had to note down ten words and three phrases dictated by the investigator.
14. Naming of body parts (written): The subject had to understand and indicate eight parts of the body.
15. Written naming of actions: The subject had to graphically produce phrases referring to six pictures arranged on six different boards shown by the investigator.
16. Repetition of numbers: The subject had to repeat 10 items referring to numbers.
17. Reading of numbers: The subject had to read 10 numbers.

All tasks in the test were awarded one point for each correct answer.

The data from all 17 subtests underwent pertinent statistical analysis, including variance analysis (ANOVA), taking a significance level of 5% and using percentile distributions.

## RESULTS

The results relating to the profile of the normal subjects who underwent the Montreal Toulouse (Modified MT Beta-86) tests are presented below.

Table 1 compares the normal subjects’ performance according to schooling level. It shows that there were statistically significant differences between the schooling bands in relation to oral comprehension, reading, graphical comprehension, naming, lexical availability, dictation, graphical naming of actions and number reading tasks.

Although the oral comprehension performance was found to be similar for the groups with schooling levels of 1-4 years and 5-8 years, greater performance was seen for the group with schooling levels of nine years and over. No difference in performance was seen between the schooling levels of 5-8 years and nine years and over, in relation to reading, naming, dictation, graphical naming of actions and number reading tasks, while the group with 1-4 years of schooling showed lower performance. There was a difference between the groups with schooling levels of 1-4 years and nine years and over in the graphical comprehension task, and also between all three schooling groups in the lexical availability task, in which performance increased with greater schooling.

Table 2 shows the normal subjects’ performance according to age bracket. It shows that there were statistically significant differences between the age groups regarding lexical availability, such that the 17-30 year and 31-50 year groups showed similar but lower performance than in the group aged 51 years and over, which scored highest for lexical availability. A statistical difference was seen for dictation between the groups aged 17-30 years and 51 years and over.

The percentage performances of the normal subjects in the Montreal Toulouse (Modified MT Beta-86) tests are shown in Table 3, with score distributions in percentiles for subtests of these tests. The percentiles most recommended for such populations in these tests were 25, 50 and 75. The items of automatisms, copying and repetition of numbers were considered constant, and therefore no percentiles were calculated for them.

**Table 1.** Subjects’ performance in language assessment test according to schooling level

Subtests		Schooling (years)			ANOVA sig. F (P)
		1-4	5-8	≥ 9	
Guided interview	Mean	11.82 (0.39)	11.83 (0.38)	11.85 (0.36)	0.943
Automatisms - form	Mean	29.00 (0.00)	29.00 (0.00)	29.00 (0.00)	Not applicable
Automatisms - content	Mean	3.00 (0.00)	3.00 (0.00)	3.00 (0.00)	Not applicable
Oral comprehension	Mean	38.73 (2.41)	39.33 (2.06)	40.68 (1.25)	0.001
Repetition	Mean	32.45 (0.96)	32.17 (1.09)	32.68 (0.64)	0.103
Reading	Mean	31.41 (1.74)	32.38 (0.77)	32.68 (0.88)	0.001
Graphical comprehension	Mean	12.09 (1.23)	12.50 (0.72)	12.71 (0.63)	0.037
Naming	Mean	26.50 (2.86)	28.08 (2.43)	28.79 (1.87)	0.003
Lexical availability	Mean	14.55 (6.08)	18.67 (5.91)	23.94 (4.89)	< 0.001
Buccofacial praxis	Mean	5.77 (0.43)	5.83 (0.48)	5.94 (0.24)	0.248
Oral comprehension - parts of the body	Mean	7.91 (0.29)	7.92 (0.41)	8.00 (0.00)	0.368
Handling of objects	Mean	7.95 (0.21)	7.96 (0.20)	7.97 (0.17)	0.948
Copying	Mean	3.86 (0.47)	4.00 (0.00)	4.00 (0.00)	0.090
Dictation	Mean	9.05 (2.90)	11.83 (1.61)	12.68 (0.73)	< 0.001
Graphical comprehension - parts of the body	Mean	7.77 (0.61)	7.88 (0.34)	7.97 (0.17)	0.176
Written naming - actions	Mean	5.09 (1.02)	5.79 (0.51)	6.06 (0.74)	< 0.001
Number repetition	Mean	10.00 (0.00)	9.96 (0.20)	10.00 (0.00)	0.315
Reading of numbers	Mean	9.45 (1.06)	9.92 (0.28)	10.00 (0.00)	0.002

Data are reported as means ± standard deviation (SD). ANOVA = analysis of variance; sig = significance. F (P) ≤ 0.005.

**Table 2.** Subjects’ performance in language assessment test according to age bracket

		Age bracket (years)				ANOVA sig. F (P)
		17-30	30-50	51 and over	Total	
Guided interview	Mean	11.71 (0.47)	11.91 (0.30)	11.82 (0.39)	11.84 (0.37)	0.264
Automatisms - form	Mean	29.00 (0.00)	29.00 (0.00)	29.00 (0.00)	29.00 (0.00)	Not applicable
Automatisms - content	Mean	3.00 (0.00)	3.00 (0.00)	3.00 (0.00)	3.00 (0.00)	Not applicable
Oral comprehension	Mean	40.57 (1.87)	39.81 (1.80)	39.32 (2.24)	39.74 (2.04)	0.150
Repetition	Mean	32.86 (0.36)	32.44 (0.80)	32.32 (1.09)	32.46 (0.90)	0.172
Reading	Mean	32.79 (0.43)	32.28 (1.02)	31.97 (1.59)	32.24 (1.26)	0.119
Graphical comprehension	Mean	12.57 (0.76)	12.72 (0.63)	12.21 (1.07)	12.47 (0.89)	0.055
Naming	Mean	29.00 (1.84)	27.66 (2.55)	27.79 (2.64)	27.95 (2.50)	0.220
Lexical availability	Mean	23.71 (6.02)	21.16 (5.98)	16.85 (6.61)	19.78 (6.74)	0.001
Buccofacial praxis	Mean	5.93 (0.27)	5.91 (0.30)	5.79 (0.48)	5.86 (0.38)	0.385
Oral comprehension - parts of the body	Mean	8.00 (0.00)	7.91 (0.39)	7.97 (0.17)	7.95 (0.27)	0.476
Handling of objects	Mean	8.00 (0.00)	7.97 (0.18)	7.94 (0.24)	7.96 (0.19)	0.614
Copying	Mean	4.00 (0.00)	3.97 (0.18)	3.94 (0.34)	3.96 (0.25)	0.750
Dictation	Mean	12.86 (0.36)	11.59 (1.85)	10.64 ( 2.93)	11.42 (2.36)	0.009
Graphical comprehension - parts of the body	Mean	8.00 (0.00)	7.78 (0.49)	7.94 (0.34)	7.89 (0.39)	0.122
Naming of actions	Mean	5.93 (0.27)	5.81 (0.47)	5.53 (1.21)	5.71 (0.86)	0.242
Number repetition	Mean	10.00 (0.00)	9.97 (0.18)	10.00 (0.00)	9.99 (0.11)	0.478
Reading of numbers	Mean	10.00 (0.00)	9.91 (0.39)	9.68 (0.84)	9.83 (0.61)	0.156

Data are reported as means ± standard deviation (SD). ANOVA = analysis of variance; sig = significance. F (P) ≤ 0.005.

**Table 3.** Subjects’ performance in the Montreal Toulouse (Modified MT Beta-86) test

	Percentiles						
	5	10	25	50	75	90	95
Guided interview	11.00	11.00	12.00	12.00	12.00	12.00	12.00
Oral comprehension	33.50	35.00	38.50	40.00	41.00	42.00	42.00
Repetition	30.00	31.00	32.00	33.00	33.00	33.00	33.00
Reading	27.50	31.00	32.00	32.00	33.00	33.00	33.00
Graphical comprehension	10.00	10.00	12.00	13.00	13.00	13.00	13.00
Naming	23.00	23.00	25.00	27.00	30.00	31.00	31.00
Lexical availability	5.00	8.00	12.00	17.00	26.00	29.00	30.50
Buccofacial praxis	4.50	5.00	6.00	6.00	6.00	6.00	6.00
Naming of body parts (oral)	7.00	8.00	8.00	8.00	8.00	8.00	8.00
Handling of objects	7.00	7.00	8.00	8.00	8.00	8.00	8.00
Dictation	4.00	6.00	8.00	12.00	13.00	13.00	13.00
Naming of body parts (written)	6.50	7.00	8.00	8.00	8.00	8.00	8.00
Graphical naming (actions)	3.50	4.00	5.00	6.00	6.00	6.00	6.00
Reading of numbers	7.00	8.00	10.00	10.00	10.00	10.00	10.00

## DISCUSSION

A critical analysis of the results obtained in our study follows.

The breakdown of our sample into groups with schooling levels of 1-4 years, 5-8 years and nine years and over (Table 1) was carried out in line with the divisions stipulated by the Brazilian Ministry of Education (Ministério da Educação, MEC), corresponding to elementary education I, elementary education II and high school, respectively. Other studies conducted in countries with large sociocultural diversity have used the same schooling breakdown.^9^^,^^10^

No gender comparison was performed in the present investigation, since reports such as the studies by Diniz et al.^11^ and Mansur et al.^12^ have observed little or no difference in linguistic performance among normal subjects between genders. In addition, the tasks that showed differences in those studies were significantly different to those performed in the present study.

Neuropsychological tests are used to investigate cognitive skills, while language and cognitive skills are known to be interrelated. However, there continues to be controversy in the literature on the issue of the influence of age and schooling on subjects’ performance in neuropsychological tests. Studies by Finlayson et al.,^13^ Rosselli et al.,^14^ See and Ryan^15^ and McCurry et al.^16^ have demonstrated that performance differences may exist among normal subjects with different ages and schooling. Table 1 shows the influence of schooling and Table 2 shows the influence of age, on the normal subjects’ performance in the Montreal Toulouse (Modified MT Beta-86) test. Both age and schooling influenced their performance, thus mirroring the findings in the literature.

Furthermore, Table 1 shows that there were statistically significant differences in the subjects’ performance in the tasks of oral comprehension, reading, graphical comprehension, naming, lexical availability, dictation, graphical naming of actions and number reading, with regard to different schooling levels. These results infer that schooling changed the subjects’ performance profile in these tasks. This could be explained in terms of meta-language development and skills acquisition over the course of knowledge-building during school and academic learning.

Social and schooling differences in batteries of neuropsychological tests have been reported by Bertolucci et al.,^17^ and similarly by Pineda et al.^9^ and Radanovic et al.^8^ in language assessment tests.

Oral and graphical comprehension tasks involve comprehension of words and phrases. Earlier studies by Pineda et al.,^9^^,^^18^ Lecours et al.,^19^ Mansur et al.,^20^ Ortiz et al.^21^ and Dabrowska and Street^10^ reported statistically significant differences in comprehension tests for language assessments according to schooling levels.

The present study revealed a difference between the groups with schooling levels of 1-4 years and 9 years and over (Table 1). It is likely that the language of subjects with a schooling level of nine years or over enables them to have better understanding of, for example, sentences in the passive voice or the use of subordinate clauses. These language features are included in the syllabus content studied in Portuguese from the fifth year of schooling onwards and their use is expected in all subsequent years during the academic education process. Thus, meta-language development may partly explain why subjects with five years of schooling or more presented higher performance in this type of test. Although our study did not show any significant difference in relation to the group with 5-8 years of schooling, it did reveal a statistically significant difference in relation to subjects with schooling of nine years or over, most likely because of the longer time spent in education.

The naming skills required in picture naming tasks involve analysis and recognition of visual elements (lines, dashes, points and curves), to generate a complex visual representation of an object. The image creates a mental representation, drawing on each subject’s internal knowledge and experience. Following this process, the object is represented with the semantic system and, finally, correct phonological activation takes place. We believe that picture-naming errors occurred either due to a failure in visual analysis, when the picture did not clearly represent the object, or due to a failure in lexical activation, since answers were only deemed correct when the subject produced exactly the same name as expected by the test.

Error analysis showed that the subjects with schooling of nine years or over were better equipped to identify pictures that named a particular semantic field, and to correctly name pictures with designs that were difficult to identify, such as the black and white image of a fire that forms board number 9 in the naming test.

Some studies have found no significant age-related differences in the naming tests in normal populations, while finding that subjects with nine years of schooling or more presented better performance in the naming tasks during the assessment.^21^^,^^22^

The lexical availability test is also known by some authors as the semantic verbal fluency test or the fluency test in the animal category. The values of 14, 18 and 24 for the groups with schooling levels of 1-4 years, 5-8 years and nine years and over, respectively, demonstrated that the number of words evoked in the animal category increased with schooling (Table 1). Many studies on tests involving semantic categories have found that such tests are influenced by schooling levels.^18^^,^^22^^,^^23^ One hypothesis for this could be that schooling facilitates organization of categories and semantic subgroups.^18^

In our study, the mean score in the lexical availability task among the normal healthy subjects was approximately 19 (Table 1). This was close to the mean scores of 17.93 found by Nitrini et al.,^24^ 20.3 by Radanovic and Mansur^23^ and 21.3 by Radanovic et al.,^8^ in studies that did not include illiterate subjects or those with less than four years of formal education.

However, the mean found in our study was greater than the values reported in several similar studies. For instance, the study by Bertolucci et al.^17^ reported a mean of 15 for verbal fluency while Brucki et al.^25^ found a mean of 13.84 animals per minute.

The tasks of dictation, graphical naming of actions and reading of numbers call for more extensive experience, acquired through schooling. Therefore, individuals with five years of schooling or more performed better (Table 1). Our study identified differences in these tasks according to schooling level (Table 1), and our findings are in agreement with those of Radanovic et al.^8^ The study by the latter authors involved applying the Boston test, and it also found differences according to schooling, in relation to the tasks of naming by visual comparison (written task based on pictures), reading of paragraphs and sentences and first-level dictation (word dictation). Their results clearly showed that exposure to formal schooling promotes development in these skills.

Table 2 shows that the 17-30 year and 31-50 year age groups differed from the group aged 51 years and over, in relation to the lexical availability task, such that the scores declined with increasing age. Analysis on the schooling levels presented by the subjects aged 51 years and over revealed that most of them had received less than four years of education, and that the sample in question was relatively small. These two factors may have influenced the statistical difference found. The same pattern was repeated in relation to the dictation task.

The studies conducted by Finlayson et al.,^13^ See and Ryan^15^ and Radanovic et al.^8^ showed that age influenced the linguistic performance in their samples. The populations investigated in these studies comprised not more than around 100 subjects.

Brucki et al.,^25^ who studied a population of over 300 subjects, found no differences in performance according to age, in relation to the fluency test on animal categories. Moreover, the study by Nitrini et al.^24^ found no decrease in verbal fluency in the animal category task with aging: their sample was divided into 10-year age brackets, in which no significant drop in performance was seen until the eighth decade.

Our study population consisted of 80 subjects who presented age influence regarding both lexical availability and dictation tasks (Table 2). From reviewing the literature, it was seen that age had an influence in populations of less than 100 subjects, whereas larger populations did not show any such influence. Therefore, we believe that the number of subjects studied may influence the results according to age. Another variable that could not be controlled for, particularly in the lexical task, was individuals’ prior experience, which can also influence individual performance.

Table 3 shows the score distribution in percentiles for each subtest of the Montreal Toulouse (Modified MT Beta-86) test. The percentiles represent measurements of the relative position of findings, in relation to the remaining values. Since the population totaled 80 subjects, the percentiles most recommended were 25, 50 and 75, for each of the Montreal Toulouse (Modified MT Beta-86) tasks. This table shows only small numerical variations among the percentiles for each subtest, except for the item of lexical availability. The percentiles described in Table 3 may serve as reference parameters for assessments on individuals with language disorders, using the Montreal Toulouse (Modified MT Beta-86) test.

Critical analysis on the results from our study allows us to conclude that the Montreal Toulouse (Modified MT Beta-86) test is sensitive to demographic variables, particularly those referring to schooling. The test significantly differentiated the normal subjects’ performance in subtests on oral comprehension, reading, graphical comprehension, naming, lexical availability, phrase and word writing, graphical naming of actions and reading of numbers.

We believe that our study presents parameters that can be used during language assessments on patients with brain damage who are evaluated using the Montreal Toulouse (Modified MT Beta-86) test.

## CONCLUSIONS

We found that schooling and age influenced the language abilities among normal adults. These variables should be taken into account during language assessments on patients with brain damage.
